# Predictors of Loss to Follow-Up among HIV-Infected Adults after Initiation of the First-Line Antiretroviral Therapy at Arba Minch General Hospital, Southern Ethiopia: A 5-Year Retrospective Cohort Study

**DOI:** 10.1155/2021/8659372

**Published:** 2021-11-11

**Authors:** Mathewos Alemu Gebremichael, Mekdes Kondale Gurara, Haymanot Nigussie Weldehawaryat, Melkamu Merid Mengesha, Dessalegn Ajema Berbada

**Affiliations:** School of Public Health, College of Medicine and Health Sciences, Arba Minch University, P.O. Box: 021, Arba Minch, Ethiopia

## Abstract

**Background:**

Loss to follow-up (LTFU) from antiretroviral therapy (ART) reduces treatment benefits and leads to treatment failure. Hence, this study was aimed at determining the incidence of loss to follow-up and predictors among HIV-infected adults who began first-line antiretroviral therapy at Arba Minch General Hospital.

**Methods:**

We carried out an institutional-based retrospective cohort study, and data were collected from the charts of 508 patients who were selected using a simple random sampling technique. All the data management and statistical analyses were conducted using STATA version 14. Cumulative survival probability was estimated and presented in the life table, and the Kaplan-Meir survival curves were compared using the log-rank test. The Cox proportional hazard model was used to identify the independent predictors.

**Results:**

We followed 508 patients for 871.9 person-years. A total of 46 (9.1%) experienced loss to follow-up, yielding an overall incidence rate of 5.3 (95% CI: 3.9-7.1) per 100 person-years. The cumulative survival probability was 90%, 88%, 86%, and 86% at the end of one, two, three, and four years, respectively. The predictors identified were age less than 35 years (adjusted hazard ratio (aHR = 1.96; 95% CI: 1.92-4.00)), rural residence (aHR = 1.98; 95% CI: 1.02-3.83), baseline body weight greater than 60 kilograms (aHR = 2.19; 95% CI: 1.11-4.37), a fair level of adherence (aHR = 11.5; 95% CI: 2.10-61.10), and a poor level of adherence (aHR = 12.03; 95% CI: 5.4-26.7).

**Conclusions:**

In this study, the incidence rate of loss to follow-up was low. Younger adults below the age of 35 years, living in rural areas, with a baseline weight greater than 60 kilograms, which had a fair and poor adherence level were more likely to be lost from treatment. Therefore, health professionals working in ART clinics and potential stakeholders in HIV/AIDS care and treatment should consider adult patients with these characteristics to prevent LTFU.

## 1. Background

Acquired immune deficiency syndrome (AIDS) is the major global public health problem that affected an estimated 36.9 million people with a global adult prevalence of 0.8% in 2018 [1]. An estimated 66% of the global burden was in sub-Saharan Africa, and of these, 19.6 million were living in East Africa [1].

In Ethiopia, the gradual decline in new HIV infections in adults was reported by the Ethiopian Public Health Institute (EPHI) HIV-related estimates and projections from 13,394 for the year 2016 to 11,613 in 2019 [2]. A total of 21.7 million people living with HIV (PLWHIV) were receiving antiretroviral therapy (ART), globally [1]. According to EPHI, in urban Ethiopia, 98.6% of adults are using ART from 2017 to 2018 [3].

Effective ART utilization controls and prevents the transmission of HIV and increases the life expectancy of HIV-infected individuals by reducing the mortality rate secondary to HIV [4]. ART had a significant contribution to the fall in new HIV infections by 37% and deaths related with AIDS by 45%, according to the World Health Organization (WHO) 2018 and Joint United Nations Programme on HIV/AIDS (UNAIDS) 2019 global HIV/AIDS epidemic report [5, 6]. And also, people on ART achieving 90% sustained viral suppression were targeted according to the United States Agency for International Development (USAID) [7]. But, loss to follow-up (LTFU) is a great challenge in achieving this target [8], and also, it was highly associated with early death [9].

In ART service utilization, loss to follow-up (LTFU) refers to a patient who had no contact for three consecutive months or longer after the last appointment for antiretroviral (ARV) refills [10]. Despite the efforts to reduce the rate of LTFU among HIV patients who are put on ART follow-up, it continued to be a challenge in HIV/AIDS care with an incidence rate ranging from 6.48 per 1000 person-months to 26.7 per person-years [11, 12].

According to the reports of different studies, the magnitude of LTF among patients living with HIV was estimated to be high. The report of the systematic review in sub-Saharan Africa showed an estimated LTFU from 20% to 40% [13], 4.1% in Asia, 21.8% in western Africa [14], 26.7 per 100 person-years in Uganda [12], and 23.9% in Nigeria [15]. In Ethiopia, a country in Eastern Africa, the incidence of LTFU varies from 0.05 per 100 person-years to 10.9 per 100 person-years across different settings [11, 16–23].

Loss of follow-up from ART service has a great negative impact on HIV patients. It can negatively affect the immunological benefits of ART and increase AIDS-related morbidity, mortality, and hospitalization, and it also results in serious consequences such as drug toxicity, treatment failure due to poor adherence, and drug resistance. It also poses a serious challenge to program implementers and constitutes an inefficient use of scarce resources like treatment [24–28]. Besides, loss to follow-up affects the performance of the 90–90–90 target that aims at achieving 90% of virally suppressed patients on ART and this is since an interference to ART follow-up lowers the success of the treatment of ART and thus leads to a decrease in the number of CD4 cell counts and increases the number of viral counts [29].

Different studies revealed that LTFU from the ART service is associated with baseline sociodemographic factors and clinical and treatment-related factors. From the sociodemographic variables: young age [16, 21, 30], male sex [18, 19, 31–33], high educational status [30, 34], single marital status [10, 35, 36], self-employed-occupational status [28, 33], rural residence [22, 33], not disclosing their status to any one [19, 34], distance from health facility over 5 kilometers [10, 11], not having a primary caregiver [11, 37], were reported predictors. Furthermore, clinical-, treatment-, and behavioral-related factors like baseline CD4 count < 200 cells/*μ*L [18, 20, 33], WHO clinical stages III and IV [21, 22, 33], taking AZT-3TC-NVP ART regimen at the start [16, 38], having an opportunistic infection at enrolment [18, 20, 39], bed-ridden and ambulatory baseline functional status [18, 20, 40], not receiving cotrimoxazole and isoniazid preventive therapy at the start [16, 20], low body mass index (BMI) (<18.5 kg/m^2^) [20], high viral loads [17], suboptimal (fair/poor) adherence level [20, 33], substance use [20], next appointment not recorded [19], and having a cell phone [22] were reported predictors. However, these factors vary from place to place in HIV patients [20].

Though few studies were done in Ethiopia, the incidence rate of loss to follow-up and predictors has not been investigated in this study setting. Therefore, this study assessed the level of incidence of loss to follow-up and its predictors among adult HIV patients on ART in Arba Minch General Hospital. The results of this study will provide necessary inputs for policymakers and program planners who are working at different levels of HIV/AIDS control programs. Besides, early identification of the problem is important to know the vital intervention areas and improve the lives of people living with HIV (PLHIV) through improved viral suppression. In resource-limited settings like Ethiopia, evidence-based interventions that prevent the loss to follow-up will improve treatment outcomes and adherence in a cost-effective approach.

## 2. Methods and Materials

### 2.1. Study Design and Setting

We conducted an institutional-based retrospective cohort study in Arba Minch General Hospital. Arba Minch town, the capital of the Gamo zone, is located in Southern Ethiopia about 444 kilometers away from Addis Ababa, the capital city of Ethiopia. Arba Minch General Hospital provides free diagnosis, treatment and monitoring, and follow-up for HIV patients, and nowadays, 1,699 adult HIV patients are actively following AR. This study included those enrolled from January 1, 2014, to December 31, 2018. Treatment and follow-up were based on the national FMOH ART treatment guidelines for HIV infections in adults and adolescents [41].

### 2.2. Population

All HIV-infected adults, aged 15 years and above, who were registered for HIV/AIDS care and put on ART from January 1, 2014, to December 31, 2018, in the Arba Minch General Hospital ART clinic, were the study population. Patients who initiated ART, but without at least one ART follow-up visit, with an incomplete patient record and those who transferred in from another facility were excluded from the study.

### 2.3. Sample Size Determination and Sampling Technique

The sample size was calculated by using STATA version 14 based on the following assumptions: 5% alpha, 80% power, allocation ratio 1, and adjusted hazard ratio 1.3 for CD4 count < 200 cells/*μ*L from the previous study conducted in Gondar, Northern Ethiopia, [18], and by adding 10%, the final sample size was five hundred eight. These samples were reached by a simple random sampling technique specifically by a computer-generated random number method. The medical registration number of patients obtained from the hospital electronic database was used as a sampling frame after excluding 106 patients who fulfill exclusion criteria.

### 2.4. Study Variables

In this study, the dependent variable was the LTFU from ART service utilization, whereas sociodemographic factors such as sex, age, education level, marital status, occupation status, residence, disclosure status (disclosed HIV status to their partner, family, or other relatives/not), substance use; treatment-related factors such as eligibility criterion (test–start/not), initial ART regimen type, comedication, treatment duration, drug side effects, number of pills per day, cotrimoxazole prophylactic treatment (CPT) at the start of ART, and the level of adherence; and clinical and immunological-related factors like baseline body weight, WHO clinical stage, CD4 count, opportunistic infections (OIs) at the start of an initial regimen, comorbidity other than OIs, baseline ALS, HGB, creatinine, and functional status were considered as the independent variable.

### 2.5. Operational Definitions

This study considered LTFU when a patient is not seen at the ART clinic for at least 90 days or 3 months after the last missed appointment, but not transferred out from the facility to another facility or dead [19, 20, 42–44]. The time to LTFU was the time interval between the dates of ART initiation and the last missed appointment, and censored are patients who died while on treatment, changed the first-line regimen, or completed the follow-up period without developing the event (LTFU) [19, 20, 42]. The following ratings to patient adherence to ART were made by the attending physician or nurse: good (95% of pills were taken: missed only 1 dose out of 30 or 2 doses out of 60), fair (85–94% of pills were taken: missed 2–4 doses out of 30 or 4–9 doses per 60), and poor (less than 85%: missed more than 5 doses out of 30 or greater than 10 doses out of 60) [20, 42]. The functional status of participants was assessed and labelled as working when an individual was able to the perform usual work inside or outside the home, ambulatory when able to perform only the activities of daily living (ADL), and not able to work or bedridden when not able to perform ADL [20]. In this study, also substance abuse was measured as any history of harmful or hazardous use of psychoactive substances, including alcohol and illicit drugs [20]. A card was considered incomplete when the indicator of the dependent variable and/or 20% of the independent variables were not registered in the chart [45].

### 2.6. Data Collection Tool and Procedures

The data collection tool was prepared by reviewing various kinds of literature in the English version that included sociodemographic characteristics, clinical characteristics, treatment-related characteristics, and the information related to the time of ART initiation and end date of LTFU and censored. The data were collected by three BSC nurses who had previous exposure to data collection and training on comprehensive HIV care and supervised by two master's holders in public health who had experience in data management.

### 2.7. Data Quality Assurance

To assure the quality of data, the data extraction format was designed carefully and we appropriately recruited data collectors who had experience in data collection. And also, training was given for data collectors and supervisors. A pretest was performed on 5% of the populations before data collection in the same setting in Arba Minch General Hospital because the source of data was secondary. Intensive supervision was done on a daily basis by the principal investigator and supervisors during the whole period of data collection.

The principal investigator reviewed a random sample of registration forms to confirm the reliability of data before the data collection period, and the investigator and supervisors made random crosschecking for the completeness, accuracy, and consistency of data at the end of each day, and accordingly, corrective discussion was made with all data collectors. To minimize errors and take corrective actions, remarks were given during morning times. Then, the data were checked for completeness and consistency and coded and entered into the computer using EpiData version 4.6.

### 2.8. Data Processing and Statistical Analysis

Data were cleaned, edited, coded, and entered into EpiData version 4.6 and exported to STATA version 14 for analysis. Exploratory data analysis was carried out to check the missing values. Results are presented using numerical summary measures, frequency, and percentages and also using survival curves and tables. The incidence rate of loss to follow-up was calculated by dividing the numbers who experienced the loss to follow-up in the overall follow-up period by person-time at risk contributed throughout the observation period. A life table was used to estimate the cumulative survival time in each time interval. The Kaplan–Meier survival curve together with the log-rank test was used to estimate the median time and to compare the overall survival experience of two or more categories of variables.

A Cox proportional hazards model was applied to identify the factors that predicted LTFU. Bivariable Cox regression analysis was used to identify candidate variables in the multivariable Cox regression. Those variables with *p* ≤ 0.25 levels in the bivariable analysis which had improved model adequacy compared with the null model were entered into the multivariable Cox regression analysis. Model adequacy was checked via the maximum likelihood with the assumption of the higher is the better. The backward variable selection method was applied to get significantly associated predictors, and the adjusted hazard ratio (aHR) with corresponding 95% confidence interval (CI) and the *p* value were used to assess the strength of association and statistical significance, respectively. The statistical significance was considered at a *p* value < 0.05. The reasonability of proportional hazard assumptions was tested graphically and by using the Schoenfeld residuals test (phtest) by using a post estimation command “*estat phtest*” and “*estat phtest, detail*” for the final model and each variable, respectively. In the final model, the global test of the Schoenfeld residuals test gave *χ*^2^(df) = 13.15 with a *p* value = 0.4361; this supports the null hypothesis which states that hazards are proportional and the iteration of log-likelihood was −266.084 in the first step and −228.4955 in the last iteration of log-likelihood with LR *x*^2^ = 75.18 and a *p* value < 0.001.

## 3. Results

### 3.1. Sociodemographic Characteristics

A total of 508 charts of HIV patients were reviewed and analyzed, giving a 100% response rate. The mean age at initiation of ART ± SD (standard deviation) was 35.1 ± 8.2 years. A majority, 219 (43.1%), were in the age groups between 25 and 34 years. A total of 289 (56.9%) were females. Regarding the educational status of the patients, a majority, 195 (38.4%), completed the primary education level and about 63 (12.4%) had tertiary and above educational statuses. A total of 375 (73.8%) were urban dwellers but the rest were rural dwellers. A total of 485 (95.5%) disclosed their HIV status to either their family or other relatives, but the rest did not disclose their HIV status (Table 1).

### 3.2. Baseline Clinical-, Immunological-, and Treatment-Related Characteristics

A total of 238 (46.9%) patients started ART by test and start eligibility criteria, but 270 (53.1%) were not by test and start. Initially, a majority, 498 (98.1%), of HIV patients have prescribed a combination of tenofovir (TDF) + lamivudine(3TC) + efavirenz (EFV), followed by tenofovir (TDF) + lamivudine(3TC) + nevirapine (NVP), zidovudine (AZT) + lamivudine(3TC) + efavirenz (EFV), and zidovudine (AZT) + lamivudine(3TC) + nevirapine (NVP). A majority of patients, 491 (96.7%), started by taking one ART pill per day whereas the rest started by taking more than one ART pill and 284 (55.9%) of the patients took CPT prophylaxis at the start of ART (Table 2).

Of all study participants, a majority, 271 (53.4%), were in WHO clinical stage I at the initiation of ART and 395 (77.8%) of the participants were on a working functional status at baseline. The median weights of the study participants at the initiation of ART and at the end of the follow-up period were 55.0 kg (interquartile range (IQR): 48–60) and 57.6 kg (IQR: 52–60), respectively. The median CD4 count at initiation of ART was 281.5 cell/mm^3^ (IQR: 144.5–407.0) and it was 457.3 cell/mm_3_ (IQR: 400–485.8) at the end of the follow-up period, and above half, 306 (60.2%), of study subjects started ART at a HGB level of between 10 and 12.9 g/dL. A total of 84 (16.5%) of the study participants have been diagnosed with tuberculosis (TB) at the initiation of ART, and about 85 (16.73%) had an opportunistic infection (OIs) other than TB. From the total study subjects, about 157 (30.9%) experienced at least one ART drug side effect (Table 2).

### 3.3. The Incidence Rate of LTFU

A total of 508 patients on ART were followed for a minimum of 0.5 and a maximum of 54.1 months of the follow-up period. The total follow-up period was 10,462.5 person-months (871.9 person-years) of observation, and the median follow-up period was 16.1 months (IQR; 5.9–33.6). At the end of the follow-up period, a total of 46 (9.1%) patients experienced loss to follow-up, 16 (3.2%) died and were defaulters, 55 (10.8%) transferred out, 292 (57.48%) were kept on an initial regimen, and 99 (19.5%) of patients changed their initial regimen. Hence, the overall incidence of loss to follow-up was estimated to be 5.3 (95% CI: 3.9-7.1) per 100 person-years of observation (PYs). From the total incidence rate of loss to follow-up, 4.0/100 PY, 40.5/PY, and 54.1 attributed to good, fair, and poor levels of adherence, respectively (Table 3).

### 3.4. Time until the LTFU

**Figure 1:**
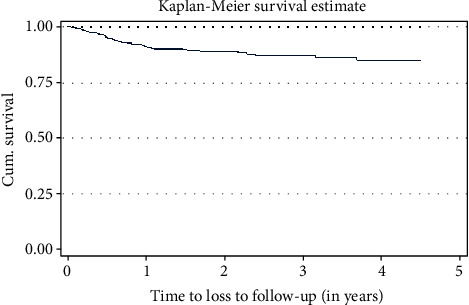
The Kaplan–Meier estimate of the loss to follow-up among adult patients attending the ART clinic at Arba Minch General Hospital, Southern Ethiopia, 2019.

**Figure 2:**
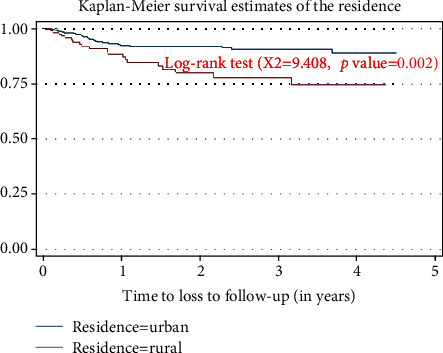
The Kaplan–Meier estimate of the loss to follow-up in resident categories among adult patients attending the ART clinic at Arba Minch General Hospital, Southern Ethiopia, 2019.

In this study, the probability of cumulative survival was 90%, 88%, and 86% at the end of one-two, three, and four years, respectively. The overall Kaplan–Meir survival function estimate showed that most of the loss to follow-up from the initial ART regimen occurred in the earlier year of ART initiation, which declined in the later years of follow-up (Table 4, Figure 1). Regarding the survival estimate difference by residence, patients in the urban areas had better mean survival time than those in the rural areas and the difference was significant (*p* value = 0.002) (Table 5, Figure 2).

### 3.5. Log-Rank Estimate of the Variables

The log-rank (Mantel-Cox) estimate of the loss to follow-up among variables was estimated for sociodemographic-, clinical-, immunological-, and treatment-related variables. Among all variables, the age, residence, level of adherence, disclosure status, regimen change, side effect, and treatment duration had a *p* value less than 0.05. There was a marginal significant difference within categories of occupational status and baseline WHO clinical staging (Table 6).

### 3.6. Predictors of Loss to Follow-Up

To assess the predictors of loss to follow-up, both bivariable and multivariable cox regression analyses were applied. From bivariable cox regression analysis, predictors with *p* values less than or equal to 0.25 were considered as the candidate variables for multivariable cox regression analysis. These were age, residence, occupational status, disclosure status, substance use, baseline weight, baseline functional status, level of adherence, baseline WHO stage, and the eligibility criteria at the start of ART (test start). In multivariable Cox regression analysis, the age, residence, level of adherence, and baseline weight were significantly associated predictors of LTFU. However, variables like a history of TB, OIs, history of cotrimoxazole prophylactic therapy, baseline CD4 count, WHO stage, and disclosure status were not significantly associated with loss to follow-up in ART (Table 7).

In this study, patients attending ART clinics who had the age less than 35 years had approximately two times higher risk of loss to follow-up when compared with those patients whose age were greater than 35 years (aHR = 1.96; 95% CI: 1.92-4.00). Regarding the residence of ART patients, those patients who were from rural areas had approximately two times higher risk of loss to follow-up as compared with counterparts (aHR = 1.98; 95% CI: 1.02-3.83). Keeping other variables constant, baseline body weight had also a significant association with the incidence of loss to follow-up. Those ART patients with baseline body weight greater than 60 kilograms had a 2.19 times higher risk of LTFU as compared with counterparts with a baseline body weight less than 60 kilograms (aHR = 2.19; 95% CI: 1.11-4.37). The hazard of LTFU was also higher among adult ART patients who had fair and poor levels of adherence. Those patients who had a fair level of adherence were 11.5 times at higher risk of loss to follow-up as compared to those with a good level of adherence (aHR = 11.5; 95% CI: 2.10-61.10), and also, patients with a poor adherence level were 12.03 times at higher risk of loss to follow-up when compared with those patients with a good level of adherence (aHR = 12.03; 95% CI: 5.4-26.7) (Table 7).

## 4. Discussion

Loss to follow-up is the major difficulty to treatment success and it complicates the evaluation of HIV care and treatment programs [12]. This study assessed the incidence rate of loss to follow-up and its predictors among adult HIV patients who started ART. In this study, 46 (9.1%) adult HIV patients experienced loss to follow-up and the overall incidence rate of loss to follow-up was 5.3 (95% CI: 3.9-7.1) per 100 person-years of observation (PYs). This finding is consistent with the study conducted in Debre Markos, Ethiopia, which reported 3.7 per 100 person-years [22].

But, this finding is lower than the study conducted in different parts of Ethiopia, in Gondar, Ethiopia, 12.26 per 100 person-years [20], in Jigjiga town, Eastern Ethiopia, 26.6 per 100 person-months [19], in Gondar Hospital, Ethiopia, 31.4% [18], in Pawi Hospital, Ethiopia, 11.6 per 100 person-years [21], in Gondar Hospital, Ethiopia, 10.90 per 100 person-years [16], and in Woldia, Ethiopia, 8.36 per 100 person-years [17], and higher than the study conducted in Mizan-Tepi University, Ethiopia, 8.8 per 1000 person-months [23] and in Southern Ethiopia 6.48 per 1000 person-months [11]. Besides, the finding of this study is also lower than the report of loss to follow-up of adult HIV patients from the treatment program from various settings out of Ethiopia, in Mbarara, Uganda, 22.85% with 16%, 30%, and 39% at 1, 2, and 3 years of initiation of treatment, respectively, and Southwestern Uganda, 26.7 per 100 person-years, similar setting in 24.6%, Masaka, Uganda, 7.5 per 100 person-years [12, 39, 46, 47], and in Southeastern Nigeria Hospital 23.9% [15].

This variation in the incidence rate might be due to the differences in setting, time variation, the difference in the follow-up period, health-seeking behaviors, and difference in the sociodemographic characters of the study participants, and the commencing of test and start ART criteria may be the other possible reason; in this study, 46.9% patients initiated their treatment by test and start eligibility criteria as compared to those of other studies. The lower incidence rate of this study from many reports that are listed above also might be due to an improvement in ART adherence, tracing back of HIV patients who lose their follow-up, and early initiation of treatment has been adapted recently to get the expected good clinical outcomes and improve the quality of life of HIV patients ultimately [48].

Regarding the characteristics of cohorts, in present study, a majority (77.8%) of study participants were in working functional status at baseline, and also, about (53.4%) were in WHO clinical stage one, (60.2%) having a hemoglobin level between 10 and 12.9 g/dL, and the median CD4 count at baseline was 281.5 cell/mm3. And also, only 16.5% of the study participants had been diagnosed with tuberculosis at the start of ART and about 16.73% had another opportunistic infection when compared with the previous studies. This all might make the lowest level of LTFU in present study. This recent study can reflect the implementation of different stakeholders' strategies.

In this study, from the total patients who experienced loss to follow-up, a majority, 34 (93.9%), lost from ART service within the first year of treatment initiation. The probability of cumulative survival was 90%, 88%, and 86% at the end of one, two, three, and four years, respectively. In this study setting, we found that a progressive decrease in the incidence rate of LTFU of HIV patients within each year after initiation of ART. This finding is in agreement with the study finding from Jigjiga town, Ethiopia [19]. This progressive decrease might be because the Ethiopian health care delivery approach decentralizes the care from referral centers to primary health care facilities; this makes the clinics closer to patients' homes requiring them to travel less and minimizes transport costs. This proximity can also make tracing more easy and feasible from decentralized clinics. Another possible reason might be the enhancement of community outreach services, such as involving community health workers, recording detailed information of each patient following ART clinics, and probably expanding the peer outreach program [49, 50].

In this study, patients whose age less than 35 years had a 1.96 times higher risk of loss to follow-up when compared with those patients whose age is above 35 years. In agreement with this study, younger age groups were more likely to lose to follow-up from ART service as compared to older age categories in the report of studies in Gondar, Ethiopia [16], Pawi General Hospital, Northwest Ethiopia [51], in South Africa [52], Nigeria [36], Oromia region, Ethiopia [33], and India [35]. The possible reason might be that the younger age group could be more mobile than the older one and may face the fear of discrimination and stigma. The other possible reasons might be immaturity in analytical thinking and particular challenges associated with young age.

Regarding the residence of HIV patients following the ART clinic, those patients who had rural residence were 1.98 times at higher risk of loss to follow-up as compared with their counterparts. This result is congruent with different studies done in the Oromia region, Ethiopia [33]. This might be because patients from the rural area may face a problem of accessibility or distance from treatment centers, transport-related costs, low level of awareness on the benefit and risk related to the adherence of treatment, and social stigma related to a disease HIV/AIDS. In contrary to this finding, a study done in Debre Markos, Ethiopia, reported that the risk of LTFU among rural residents was reduced by 40% when compared with urban residents [22]. The possible reason given is that study were patients coming from rural areas preferred to have ART follow-up in the nearby hospitals; this is because rural residents are stable or permanent residents compared to urban residents and due to the scarcity of money; they may not use the self-referral system to other healthcare institutions when compared to urban residents [22].

Furthermore, baseline body weight had also a significant association with loss to follow-up in this study. Those ART patients with baseline weight greater than 60 kilograms were 2.19 times at higher risk of LTF as compared with those with baseline weight less than 60 kilograms. The possible reason could be, even though BMI was not computed in this study, those patients with higher body weight might face noncommunicable diseases which have been associated with overweight and obesity and this could affect their retention in care, and due to these chronic noncommunicable diseases, they might have a suboptimal (fair/poor) adherence level which is a predictor of LTFU in the present study. Another reason might be that patients with normal body weight would like to maintain their good wellbeing (i.e., they do not want to lose) by taking ART so that they stay longer in the program when compared to those whose body weight is not normal [38].

The adherence status had a statistically significant association with the incidence rate of LTFU. The risk of LTFU was higher among adult ART patients with fair and poor levels of adherence. Those patients who had a fair level of adherence were 11.5 times at higher risk of loss to follow-up as compared to those with a good level of adherence, and patients with a poor adherence level were 12.03 times at higher risk of loss to follow-up when compared with those patients with a good level of adherence. This finding is in agreement with a study done in Gondar, Ethiopia [20], Oromia region, Ethiopia [33]. These two studies merged fair and poor adherence statuses and defined it as suboptimal adherence. In both studies, patients with the suboptimal level of adherence were at a higher risk of loss to follow-up when compared to the good or optimal level of adherence. The possible explanation might be that those patients with fair and poor/suboptimal adherence levels may have different sociodemographic, economic, and clinical problems that affect their adherence initially which further affect their retention in HIV/AIDS care.

Variables like alcohol/drug use and prescription of many pills/day were not statistically significant in this study. The possible reasons might be that the number of substance users in present study was only 1.5% and those patients who were receiving two and more pills per day were only 0.3%.

As limitations, due to the nature of the secondary source of data, some missed variables like the BMI, status of mental illness, stigma and discrimination, viral load, distance from the health facility, cell phone possession, and having a caregiver might be predictors. In addition to these, charts with an incomplete record of 20% and transferred outpatients were excluded; this might lead to underestimating or overestimating the incidence rate of loss to follow-up.

## 5. Conclusions

In this study, the incidence rate of loss to follow-up was estimated to be low when compared with a majority of previous studies. A majority of loss to follow-up occurred within the first year of ART initiation. Younger adults below the age of 35 years, living in rural areas, had a baseline body weight greater than 60 kilograms, had a fair and poor adherence level, and were more likely to be lost from treatment. Therefore, health professionals working in ART clinics and potential stakeholders in HIV/AIDS care and treatment should give emphasis for above characteristics. They should give health education about the effects of LTFU and the benefits of retention in the ART program for adult patients below the age of 35 years, visits to ART clinic from the rural (distant) areas, having baseline body weight above 60 kilograms, and having a suboptimal (fair and poor) adherence level. Furthermore, a prospective cohort study needs to be conducted and a qualitative study should be applied to obtain information from LTFU patients themselves by tracing them and the health service quality and satisfaction level of patients need to be addressed.

## Figures and Tables

**Table 1:** Baseline sociodemographic characteristics of HIV-positive adults at the initiation of ART at Arba Minch General Hospital, Southern Ethiopia, 2019 (*n* = 508).

Characteristics	Categories	Frequency (*n*)	Percentage (%)
Age (years)	15–24	31	6.1
	25–34	219	43.1
	35–44	183	36.0
	≥45	75	14.8
Sex	Female	289	56.9
	Male	219	43.1
Marital status	Married	298	58.7
	Divorced	90	17.7
	Single	67	13.2
	Widowed	53	10.4
Educational status	Primary	195	38.4
	Secondary	138	27.2
	Not educated	112	22.1
	Tertiary and above	63	12.4
Religion	Orthodox	308	60.6
	Protestant	173	34.1
	Muslim	20	3.9
	Others^∗^	7	1.4
Occupation	Unemployed	378	74.4
	Employed	130	25.6
Residence	Urban	375	73.8
	Rural	133	26.2
Substance use	Yes	76	14.9
	No	32	85.1
Disclosure status	Disclosed	485	95.5
	Not disclosed	23	4.5

^∗^ = Catholic religion followers, Traditional believers.

**Table 2:** Baseline clinical-, immunological-, and treatment-related characteristics of HIV-positive adults on ART in Arba Minch General Hospital, Southern Ethiopia, 2019 (*n* = 508).

Characteristics	Categories	Frequency (*n*)	Percentage (%)
Initial regimen	TDF + 3TC + EFV	498	98.0
	AZT + 3TC + EFV	3	0.6
	AZT + 3TC + NVP	4	0.8
	TDF + 3TC + NVP	3	0.6
ART pills per a day	One	491	96.7
	Two	17	3.4
CPT at start of ART	Yes	284	55.9
	No	224	44.1
Comedication other than CPT	Yes	111	21.9
	No	397	78.2
TB at start	Yes	84	16.5
	No	424	83.5
OIs other than TB at the start	Yes	85	16.7
	No	423	83.3
Comorbidity other than OIs	Yes	11	2.2
	No	497	97.8
Functional status	Working	395	77.8
	Ambulatory and bedridden	113	22.2
WHO clinical stage	Stage I	271	53.4
	Stage II	97	19.1
	Stage III	124	24.4
	Stage IV	16	3.2
Baseline weight (in kg)	≤60 kg	384	75.6
	>60 kg	124	24.4
Baseline CD4 count (cells/mm^3^)	<100 cells/mm^3^	88	17.3
	100–199 cells/mm^3^	89	17.5
	200–349 cells/mm^3^	155	30.5
	≥350 cells/mm^3^	176	34.7
Baseline HGB (g/dL)	<7 g/dL	10	2.4
	7–9.9 g/dL	60	11.8
	10–12.9 g/dL	306	60.2
	≥13 g/dL	132	25.9
Baseline creatinine	0.00–0.59 mg/dL	93	18.3
	0.6–1.2 mg/dL	379	74.6
	>1.2 mg/dL	36	7.1
Baseline alanine transaminase (IU/L)	<7 IU/L	9	1.8
	7–56 IU/L	472	92.9
	>56 IU/L	27	5.31
Drug side effect	Yes	157	30.9
	No	351	69.1
Treatment duration	<1 year	198	38.9
	1–2 years	109	21.5
	>2 years	201	39.6

ART: antiretroviral therapy; AZT: zidovudine; CD4: clusters of differentiation; CPT: cotrimoxazole prophylaxis therapy; EFV: efavirenz; HGB: hemoglobin; IU/L: international unit per liters; kg: kilogram; TDF: tenofovir; +3TC: lamivudine; NVP: nevirapine; TB: tuberculosis; OIs: opportunistic infections; WHO: World Health Organization.

**Table 3:** Incidence rate of loss to follow-up among HIV-positive adults on ART in Arba Minch General Hospital, Southern Ethiopia, 2019 (*n* = 508).

Characteristics	Categories	Person-time (PY)	Failures (LTFU)	Rate (IR)	95% CI (IR)
Level of adherence	Good	844.8	32	0.04	0.03-0.05
	Fair	4.9	2	0.41	0.10-1.62
	Poor	22.2	12	0.54	0.31-0.95
Residence	Urban	672.9	26	0.04	0.03-0.06
	Rural	199.0	20	0.10	0.06-0.16
Baseline age	<35 years	400.4	32	0.08	0.06-0.11
	≥ 35 years	471.5	14	0.02	0.02-0.05
Baseline weight	≤60 kg	653.6	31	0.05	0.03-0.07
	>60 kg	218.3	15	0.07	0.04-0.11
Total (overall incidence rate)		871.9	46	5.3	0.039-0.071

CI: confidence interval; IR: incidence rate; LTFU: loss to follow-up; PYs: person-years; kg: kilogram.

**Table 4:** Life table on the incidence rate of LTFU among adult HIV patients at Arba Minch General Hospital, Southern Ethiopia, 2019 (*n* = 508).

Interval	Beg. total	LTFU	Withdraw	At risk	Proportion of surviving	Cum. proportion of surviving
0–1	508	34	166	425	0.92	0.92
1–2	308	7	100	258	0.89	0.90
2–3	201	3	79	161	0.88	0.88
3–4	119	2	83	77	0.86	0.86
4–5	34	0	34	17	0.86	0.86

LTFU: loss to follow-up; Beg.: beginning; Cum.: cumulative.

**Table 5:** Estimated survival time of ART patients over specific categories among adult patients attending the ART clinic at Arba Minch General Hospital, Ethiopia, 2019 (*n* = 508).

Variables	Categories	Mean survival time (95% CI)
Residence	Urban	4.15 (4.02-4.28)
	Rural	3.58 (3.27-3.89)
Age (in years)	Less than 35 years	3.83 (3.61-4.05)
	Greater than or equal to 35 years	4.23 (4.08-4.37)
Weight (in kg)	Less than 60 kg	4.07 (3.93-4.22)
	Greater than or equal to 60 kg	3.89 (3.62-4.18)
Level of adherence	Good	4.16 (4.04-4.27)
	Fair	1.08 (0.55-1.62)
	Poor	1.76 (0.91-2.61)

CI: confidence interval; kg: kilogram.

**Table 6:** The log-rank estimate of variables among adult patients attending the ART clinic at Arba Minch General Hospital, Southern Ethiopia, 2019 (*n* = 508).

Variables	Log rank estimate		Variables	Log rank estimate	
	*χ* ^2^	*p* values		*χ* ^2^	*p* values
Age (in years)	9.001	0.003	HGB	0.045	0.835
Sex	0.034	0.854	Creatinine	0.578	0.447
Marital status	0.293	0.961	ALT	0.792	0.374
Educational status	1.457	0.692	Level of adherence	76.508	<0.001
Religion	6.116	0.106	Disclosure status	6.401	0.011
Occupation status	3.676	0.055	CPT at start	0.001	0.981
Residence	9.408	0.002	Comedication	0.032	0.851
Substance use	1.751	0.186	No. of pills per day	0.041	0.840
Baseline weight (in kg)	1.596	0.206	Initial regimen	2.659	0.447
Baseline WHO stage	3.561	0.059	Regimen change	9.816	0.002
Baseline functional status	2.352	0.308	Side effect	6.424	0.011
TB at start	1.203	0.273	Treatment duration	154.68	<0.001
OIs other than TB	1.221	0.269	Treatment failure	1.620	0.203
Baseline CD4 count	0.740	0.390	EFV vs NVP based	2.329	0.128

ALT: alanine transferase; CD: clusters of differentiation; CPT: cotrimoxazole prophylaxis therapy; EFV: efavirenz; HGB: hemoglobin; kg: kilogram; NVP: nevirapine; TB: tuberculosis; OIs: opportunistic infections; WHO: World Health Organization; *χ*^2^: chi square.

**Table 7:** Predictors of loss to follow-up among adult patients attending the ART clinic at Arba Minch General Hospital, Southern Ethiopia, 2019 (*n* = 508).

Variables	Categories	LTFU	Censored	cHR (95% CI)	aHR (95% CI)
Age(years)	<35	32	218	2.53 (1.35-4.75)	1.96 (1.92-4.00)^∗^
	≥35	14	244	1	
Sex	Male	19	200	0.95 (0.53-1.71)	
	Female	27	262	1	
Residence	Urban	26	349	1	1.98 (1.02-3.83)^∗^
	Rural	20	113	2.42 (1.35-4.34)	
Educational status	No formal education	10	102	1.92 (0.53-6.99)	
	Primary	19	176	1.97 (0.58-6.66)	
	Secondary	14	124	2.10 (0.60-7.32)	
	Tertiary and above	3	60	1	
Occupation status	Employed	6	124	1	1
	Non-employed	40	338	2.26 (0.96-5.34)	1.96 (0.73-5.27)
Marital status	Single	5	62	1	
	Married	29	269	1.22 (0.47-3.16)	
	Divorced	8	82	1.21 (0.39-3.69)	
	Widowed	4	49	0.99 (0.27-3.71)	
Disclosure status	Disclosed	43	442	1	1
	Not-disclosed	3	20	4.07 (1.25-13.3)	0.93 (0.22-3.87)
Substance use	Yes	4	72	0.51 (0.18-1.41)	0.58 (0.19-1.71)
	No	42	390	1	1
Baseline weight	≤60 kg	31	353	1	1
	>60 kg	15	109	1.48 (0.80-2.75)	2.19 (1.11-4.37)^∗^
Baseline functional status	Working	41	354	1	1
	Ambulatory and bedridden	5	108	0.54 (0.21-1.37)	1.11 (0.32-3.92)
Level of adherence	Good	32	408	1	1
	Fair	2	20	9.38 (2.2-39.99)	11.5 (2.1-61.1)^∗^
	Poor	12	34	12.49 (6.3-24.8)	12.03 (5.426.7)^∗^
Baseline WHO stage	Stage 1	32	239 90 118 15	1	1
	Stage 2	7		0.65 (0.29-1.50)	0.69 (0.26-1.81)
	Stage 3	6		0.42 (0.18-1.01)	0.65 (0.23-1.89)
	Stage 4	1		0.45 (0.06-3.31)	2.14 (0.21-22.3)
Baseline CD4 count	< 200	16	161	1.31 (0.71-2.39)	
	≥ 200	30	301	1	
TB at start	Yes	4	80	0.57 (0.20-1.58)	
	No	42	382	1	
OIs other than TB	Yes	4	81	0.57 (0.20-1.58)	
	No	42	381	1	
CPT at start	Yes	27	257	1	
	No	19	205	1.01 (0.56-1.81)	
Comedication other than CPT	Yes	1	10	1.19 (0.16-8.69)	
	No	45	452	1	
Test-start	Yes	25	213	1	1
	No	21	249	0.45 (0.25-0.81)	0.84 (0.43-1.6)
Numbers of pills/day.	One	44	447		
	Two and above	2	15	0 .86 (0.2-3.57)	

^∗^Indicates a *p* value < 0.05; 1: reference category; cHR: crude hazard ratio; aHR: adjusted hazard ratio; CI: confidence interval; kg: kilogram; TB: tuberculosis; OIs: opportunistic infections; CD4: cluster of differentiation; CPT: cotrimoxazole prophylaxis therapy; LTFU: loss to follow-up.

## Data Availability

All generated and analyzed datasets are available with a reasonable request through the corresponding author.

## Abbreviations

aHR: Adjusted hazard ratio
AIDS: Acquired immune deficiency syndrome
ART: Antiretroviral therapy
CD4: Cluster of differentiation
CPT: Cotrimoxazole prophylactic therapy
EPHIA: Ethiopia population-based HIV impact assessment
HIV: Human immunodeficiency virus
IRB: Institution review board
IQR: Interquartile range
LTF: Loss to follow-up
OIs: Opportunistic infections
PLWHIV: People living with HIV
PYs: Person-years
PMs: Person-months
TB: Tuberculosis
UNAIDS: Joint United Nation Program on HIV/AIDS
WHO: World Health Organization.
